# “It’s Not Like Chasing Chanel:” Spending Time, Investing in the Self, and Pandemic Epiphanies

**DOI:** 10.1177/07308884221125246

**Published:** 2022-09-15

**Authors:** Alexandrea J. Ravenelle, Ken Cai Kowalski

**Affiliations:** 1 Sociology, 169101 University of North Carolina at Chapel Hill College of Arts and Sciences, Chapel Hill, NC, USA

**Keywords:** work passion, great resignation, precarious work, gig work, experiences of time

## Abstract

The Covid-19 pandemic has greatly impacted the labor market and given rise to the Great Resignation. Drawing on a mixed methods panel study of 199 precarious and gig-based workers, we analyze how a changing conception of free time during the Covid-19 pandemic led low-wage service workers to seek more fulfilling careers. Whereas most workers initially perceived free time in terms of opportunity costs, they later reconceived this time as enabling an investment in personal growth, moving from “spending time” making money to “investing time” in themselves. This shift in temporal experience is expressed through the adoption of a “work passion” logic and “pandemic epiphanies” that motivated respondents to seek self-affirming and potentially more lucrative work opportunities.

The rise of contractual and part-time employment has transformed experiences of time in the employment relationship by blurring distinctions between work and home life (Kalleberg & Fuchs Epstein, 2001) while increasing uncertainty around work scheduling (Schneider, 2021). Workers’ experiences of these changes are often described in terms of hysteresis, or misalignment between expectations based on past experiences and present circumstances (Ayala-Hurtado, 2021; McDonough & Polzer, 2012). These accounts document how prevailing cultural narratives about fairness and meritocratic achievement impede white-collar workers’ adaptation to precarious economic conditions. Similarly, other research shows that aspiring professionals adhere to an ideology of personal fulfillment through work that tethers them to low-paying and unstable jobs (Pagis, 2021).

In this paper, we follow DePalma’s (2021) call to examine how this “work passion” ideology can potentially lead workers to higher-paying or more stable employment. We suggest that low-wage service workers’ changing conceptions of free time during the Covid-19 pandemic led them to seek more fulfilling and lucrative careers, and may have contributed to the Great Resignation as workers leave their jobs in historic numbers (Cook, 2021; Tharoor, 2021).

Utilizing a mixed methods panel study of 199 precarious and gig-based workers, we find that early in the pandemic (April to June 2020), respondents reported that their overabundance of free time was experienced as a disruption to the conversion of time into money. The main problem facing most respondents was how to continue spending time in a way that generated income, especially if they experienced delays in receiving unemployment assistance or believed they were ineligible. During the second data collection effort (November 2020 through June 2021), which roughly coincided with the second surge of the pandemic, respondents reported using their newfound surplus time on various kinds of contemplation and self-improvement, assisted by the income security provided by weekly unemployment benefits. As respondents’ experiences of time changed from “spending time” making money to “investing time” in themselves, so did their perceptions of time: a use-mindset gave way to an investment-mindset as respondents came to value time as a resource that initiates personal growth.

Particularly for gig-based and food service workers, this process of self-reflection yielded commitments to pursue more personally fulfilling careers with better opportunities for advancement. These findings demonstrate that a sudden intensification of uncertainty followed by relative financial security enabled low-wage service workers to overcome hysteresis through a reconceptualization of their previously instrumental experience of time. Further, this process was informed by a “work passion” ideology that was previously thought to circulate primarily among creative professionals.

Drawing on Fuchs Epstein and Kalleberg (2001), we argue that this shift in the experience and subjective valuation of time, which resulted in self-reflection and desires for more fulfilling employment, may have contributed to the rise of the “Great Resignation” (Cook, 2021; Tharoor, 2021). This study also extends recent survey findings that document increased prioritization of work passion among college-educated workers experiencing job instability during the pandemic (Cech & Hiltner, 2022). We frame our research within the larger theoretical discussion of precarious work and perceptions of time.

## Theoretical Framing

### Time and Precarious Work

The growth of contingent employment has reshaped how workers’ time is structured and experienced (Fuchs Epstein & Kalleberg, 2001). Work hours have become less consistent with the increasing prevalence of ad-hoc contracts (Kalleberg, 2009, 2011) and 24-hour cycles of work in service industries (Snyder, 2016). For professionals, a higher degree of control over the labor process leads to a blurring of distinctions between work and free time, often increasing the length of the workday and straining relationships as job responsibilities are conducted at home (Kalleberg & Fuchs Epstein, 2001). For precarious workers, which includes people employed in temporary, contract-based work, and involuntary part-time work that provides limited economic and social benefits, and is covered by few labor law or regulatory protections (Kalleberg, 2009), unpredictability in work schedules may lead to increased psychological distress (Schneider, 2021).

Problems associated with inconsistent hours and blurred boundaries between work and home life are further heightened for gig workers who nominally enjoy a higher degree of control over their schedules. Though digital gig work platforms promise workers unprecedented autonomy (Ravenelle, 2019a, 2019b), flexibility often comes at the expense of stable social connections that characterize more traditional employment relations, leading to feelings of isolation and powerlessness (Glavin et al., 2021). Inconsistent contracts based on customer feedback and prevailing demand further influence when gig workers can choose to work (Ravenelle, 2019a). These issues may be particularly detrimental for the workers who are depending on gig work as a substitute for unemployment insurance during the Covid-19 pandemic, either because they were denied benefits or were unaware they qualified (Ravenelle et al., 2021).

### Navigating Precarity

Scholars have recently called for greater attention to subjective perceptions of precarious employment, especially through qualitative analysis of discourse (Vallas & Christin, 2018). As the rise of precarious work has increased uncertainty around scheduling, workers’ perceptions of these conditions have often been understood through Bourdieu's (1990, 2000) concept of hysteresis, or the misalignment between dispositions acquired through past experiences and circumstances faced in the present (Ayala-Hurtado, 2021; McDonough & Polzer, 2012). These accounts document how workers navigate intensifying job insecurity using familiar cultural narratives or values rooted in historical experience of more stable employment, thereby producing a tension between expectations and present realities that may impede successful adaptation. For instance, struggling workers with personal memories of greater job security often express nostalgia for the steady work that characterized postwar industrial capitalism in the West (Strangleman, 2007).

Two major cultural frames that have shaped worker experiences of precarity include the ideology of “work passion” (DePalma, 2021) or the “passion principle” (Cech, 2021), and conceptions of the “enterprising self” (Vallas & Christin, 2018). Widespread among precarious professionals and university students aspiring to work in cultural industries, the perception of work as a means to attain personal fulfillment has typically been cast as a contributing factor to the “self-precarisation” of young knowledge workers (Simola, 2021). A prevailing belief that hard work in pursuit of one's passions will eventually lead to a professionally and emotionally rewarding career keeps many workers optimistic about their prospects in increasingly competitive labor markets, tethering them to low-paying and unstable jobs in the process (Cech, 2021; Pagis, 2021). Devotion to personally fulfilling work can even function as a “commitment device” that leads these workers to forgo more stable jobs for fear these opportunities might weaken or change their passions (Adler, 2021). However, DePalma (2021, pp. 155–156) has recently suggested that scholars devote more attention to studying how this “work passion” ideology might also motivate workers to seek out higher-paying jobs with better working conditions.

Experiences of precarious employment have also been structured by individualized and marketized conceptions of the self. Increasingly precarious work conditions have been accompanied by a cultural logic attributing ultimate responsibility for market outcomes to individual efforts (Bauman, 2000; Beck, 1992). This “individualization” of social circumstances represents a sharp deviation from historically more communitarian values informed by religious traditions and experience with Fordist industrial production in the Western world (Lamont & Duvoux, 2014). The spread of market fundamentalism weakened belief in states’ responsibility to secure general welfare by propagating an image of human nature based on rational capital accumulation (Polanyi, 2001). The extension of market logic to varied domains of social life has also remade conceptions of the self in the image of the entrepreneur (Foucault, 2008). Studies of professional workers indicate widespread adherence to meritocratic conceptions of achievement (Ayala-Hurtado, 2021; Pagis, 2021) in combination with personal branding practices that seek to market the self as a labor-commodity (Gershon, 2017; Vallas & Christin, 2018).

These cultural frames that cast employment as both a pursuit of passion and an outcome of self-optimization for market competition comprise a new discursive logic that legitimates the precarity of increasingly flexibilized work arrangements, particularly among white-collar workers. Boltanski and Chiapello (2007, p. 169) describe this new “justificatory regime” as a “projects-oriented” discourse, observable especially in management handbooks that moralize about constant activity to increase personal “employability”. Meanwhile, self-help literature and career counselors encourage workers to interpret this ceaseless striving for employability as a journey toward discovering authentic passions, creating a culture of fixation on personal reflection and improvement that McGee (2005) terms the “belabored self.” This cultural emphasis on self-discovery as a route to employability has been observed in US white-collar workers’ understandings of unemployment, especially after the Great Recession (Damaske, 2021, Lane, 2011, Sharone, 2014; Snyder, 2016). These unemployed workers described feelings of shame and self-doubt accompanying stretches of boredom or unstructured time, and they viewed job-searching as a personal responsibility of aligning skills, goals, and passions with available opportunities—even in the aftermath of economic disasters.

### Eventful Time

Beyond the context of economic precarity, experiences of time are generally influenced by a combination of cultural meanings, social conditions, and personal agency. Adam (1994) and Mische (2014) have demonstrated that perceptions of the past and future intermingle with the rhythms of daily life to shape social and personal experience of time. At the broadest level, shared and typically unnoticed “temporal landscapes” (Tavory & Eliasoph, 2013) or “time maps” (Snyder, 2016; Zerubavel, 2003) enable individuals to locate personal trajectories within collective conceptions of time, including calendrical and clock time. Personal expectations about the future are also structured by institutionalized understandings of time, including normative conceptions of the life course, commonplace assumptions about career advancement, and taken-for-granted models of linear progression through institutions like schools (Snyder, 2016; Tavory & Eliasoph, 2013). Adam (1994, p. 120) observes that daily routines are also implicitly shaped by a “reified, internalised, and imposed” conceptualization of time “as something we can use, allocate, spend, or fill,” forming the basis for an instrumentalized relation to time.

Although experiences of time are profoundly shaped by shared and often implicit understandings, many scholars have highlighted how novel circumstances can create conditions that support transformative rethinking of life plans. Anticipated trajectories that typically feel predetermined can be consciously scrutinized when major disruptions interrupt the flow of ordinary temporal experience. Changed conditions that accompany these “events” (Wagner-Pacifici, 2017) or “moments of disjuncture” (Tavory & Eliasoph, 2013) instantiate a process of adaptation that also highlights the inadequacy of previously unquestioned expectations. Conscious recognition and revision of previously implicit beliefs about the future is most likely to occur when individuals are removed from their typical daily routines (Mische, 2014), as is often the case with unemployed workers who take the opportunity to reflect on their career plans (Damaske, 2021; Lane, 2011; Snyder, 2016). Mische (2014) terms the temporal and material conditions that support conscious evaluation of the future “sites of hyperprojectivity,” observing that alternative possibilities are difficult to conceive without the opportunity to set aside everyday responsibilities and concerns that implicitly constrain anticipation of the future.

Although changing circumstances and access to sites of hyperprojectivity facilitate new orientations toward the future, unexpected occurrences are not inherently eventful—they must first be interpreted as a significant rupture with the “ground” of mundane experience to prompt conscious reflection on prior expectations (Wagner-Pacifici, 2017). Sites of hyperprojectivity are therefore crucial for enabling deliberate “time work” (Flaherty, 2002), or the act of interpreting time through “mnemonic operations” (Zerubavel, 2003) that configure the past, present, and future in relation to the self. Sudden and unexpected occurrences can be defined as transformative events through “mnemonic cutting,” an episodic form of periodization that disconnects the event from the past. Demarcation of events often punctuates major historical eras within collective memory, but the perception of biographical events, or “turning points,” precipitates conscious reevaluation and transformation of personal trajectories. Perceiving turning points in one's own life diverges past experience from present and future aspirations (Zerubavel, 2020), preparing the self to embrace major life changes ranging from religious conversion (Snow & Machalek, 1984) to divorce (Vaughan, 1990).

Although previous studies of white-collar workers found that periods of reflection during unemployment led them to embrace the pursuit of passion as a means to improve employability in their existing fields (Lane, 2011; McGee, 2005; Sharone, 2014; Snyder, 2016), we ask whether the historic assistance provided through the CARES Act could help constitute personal sites of hyperprojectivity that enable precarious workers to move beyond the narrow goal of employability as they plan for the future. In particular, we examine whether conditions combining extreme uncertainty with unprecedented financial security could lead workers to draw on conceptions of work passion in a manner that breaks with the “self-precarisation” (Simola, 2021) and ceaseless self-optimization typically encouraged by work passion discourse and invocations of the “belabored self” (McGee, 2005). Our analysis describes workers’ changing experiences of time during the pandemic, culminating in the perception of “pandemic epiphanies,” or biographical turning points that motivated workers to reject an exclusive focus on employability in favor of seeking personal fulfillment and better work conditions.

## Methodology

The data for this mixed-methods panel study was collected in two waves. Data was collected primarily in April through June 2020, during the initial outbreak of the virus in New York City, and a time when New York was regularly described as an “epicenter” of the outbreak in the U.S (McKinley, 2020). Six interviews were conducted with Airbnb hosts in July to August of 2020, a later time frame to account for the early unknowns about whether the platform would pay hosts during widespread cancellations. Follow-up surveys and interviews were conducted from November 2020 through June 2021, to coincide with the second surge of the virus and early stages of the coronavirus vaccine roll-out.

Respondents were recruited via Facebook groups for gig workers and unemployed workers; online ads (e.g., Craigslist); and snowball sampling. Workers in the New York metropolitan area (broadly defined as Westchester, NY to Newark, NJ) who utilized gig platforms for work, or were in precarious jobs such as retail, restaurant/bar, or face-to-face creative freelance work^1^ were eligible. Participation in the second wave of data collection was limited to respondents who had participated in the first wave.

In the first round of data collection, 199 precarious workers participated including 60 creative freelancers, 33 restaurant workers, and 31 low-wage workers. Sixty gig-based workers were included from platforms such as TaskRabbit, DoorDash, Instacart, and Uber, in addition to 15 non-platform based gig workers who secured work via websites such as Craigslist. All workers had worked face-to-face before the pandemic, and 76.5 percent reported that they had experienced job or income losses due to the pandemic. In the second phase of data collection, respondents included 55 creative freelancers, 26 low-wage workers, 28 restaurant workers, and 47 gig workers, including 12 non-platform-based gig workers, an attrition rate of approximately 15 percent.

In both waves of data collection, participants completed a short online survey and participated in a respondent-directed telephone interview (Weiss, 1994). The first round of interviews sought to identify the immediate impact of the pandemic on workers: income source(s) before and during the pandemic; experiences (if any) in applying to unemployment; the pandemic's impact on their daily routine; and perceptions of how platforms or employers were handling the pandemic. The second round of interviews focused on continued life during the pandemic: how life was progressing in the pandemic; changes or continuances in sources of income/jobs; and views on the COVID-19 vaccine. Phase I interviews averaged almost 90 min, while the follow-up interviews averaged slightly under two hours.

**Figure 1.**
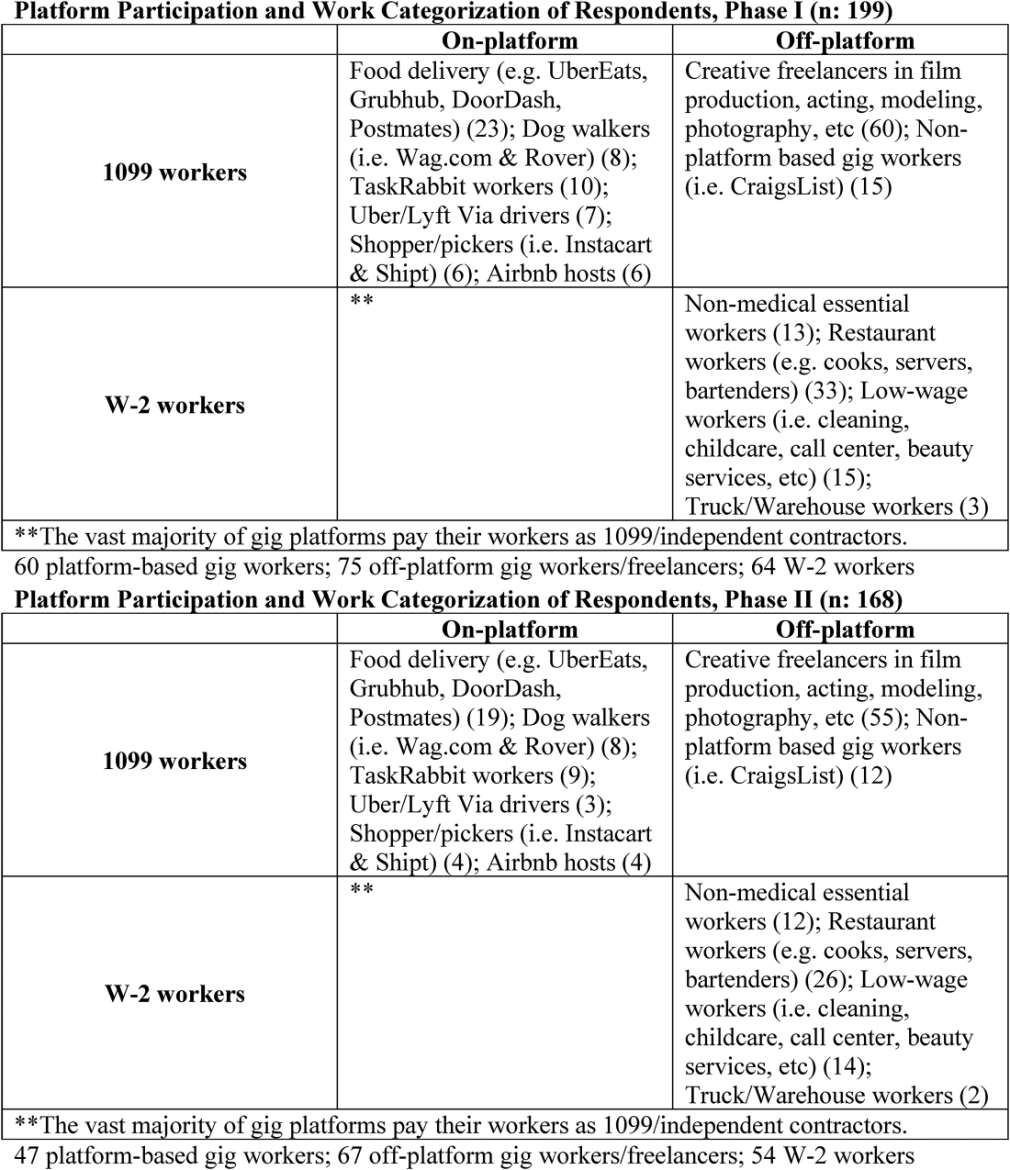
Phase I and II platform participation and work categorization of participants.

Interviews were recorded, transcribed, and coded using flexible coding (Deterding & Waters, 2021), an iterative method that is well suited for collaborative analysis of in-depth interviews. In the first round of coding, an undergraduate research assistant “indexed” the interviews at a broad level by coding by interview question (Deterding & Waters, 2021, p. 15). The second stage of the analysis involved the development and application of conceptual codes. Respondents were assigned pseudonyms, and given a $25 gift card incentive for Phase I participation, and a $50 gift card after Phase II participation (Figure 1).

Of the 199 participants, 103 (51.8%) were female, 46.2% were male, and 2% identified as gender-nonconforming or non-binary. Fewer than half (41.2%) identified as white, with 15.6% identifying as Black or African American, 14.1% as Hispanic, 14.6% as Asian, and 14.5% as multiple races. The interviewees ranged in age from 19- to 64-years-old with an average age of 33. Roughly equal numbers had an Associate's degree (9%) or a high school diploma (9%). More than a quarter had some college experience (25.1%), while 36.2% held a Bachelor's degree and 19.6% held a graduate degree or had some graduate school experience. Nearly two-thirds (60.7%) of interviewees made less than $40,000 per year, with 28.5% earning less than $20,000 per year. Almost a quarter (23.5%) of interviewees made between $40,000 and $70,000 per year before the pandemic.^2^

## Findings

In the early interviews, conducted between April and June 2020, workers broadly fell into one of three broad categories: suspended animation; preparation; or rest, reflect, and reset. These categories are “ideal types,” and the naming of these categories draws on respondents’ own descriptions of their experiences. Intriguingly, worker classification did not appear to impact the number of workers who identified as experiencing “suspended animation,” with gig-based and precarious workers alike reporting similar feelings. However, those in the “rest, reflect, and reset” category were more likely to be restaurant workers and gig-based workers, a difference that we theoretize more fully later in the findings.

### Suspended Animation: “I’ve Just Been Kind of Existing”

Respondents often discussed a sense that their sense of time had changed or lost meaning, a sensation that we call “suspended animation.” As 23-year-old Ian, an unemployed operations manager at a large media company, explained, “I am not conscious of time anymore.” Arielle, 32, an unemployed sommelier, described her average pandemic day as “Monotonous. There's just a monotony to it … the same day, the same stuff.” Melissa, 25, a film editor and delivery worker explained, “I’ve just been kind of existing.”

In some cases, respondents reported turning to screens as a way to fill the hours, often using activities that they previously viewed as enjoyable to fill the hours. “It's so boring. It was awesome at first. It's gotten so mundane,” said Hunter, 23, furloughed restaurant worker. “I just turn on my PlayStation 4 and I just play video games almost all day.” In the most extreme cases, the sense of suspended animation was seen as a response to anxiety about the pandemic and assisted with external substances. “I wake up and I’m lighting a joint. So, I keep myself sedated the whole day pretty much,” said Victor, 40, an unemployed bartender. “Because I’m worried.”

Without the daily routine of work schedules, workers often expressed a sense that they were lacking purpose. “Everything in my industry is on pause. So it's kind of difficult,” said Ian. “I’m just dying to get back into a routine. I want to wake up and feel like I actually had something to do.” Workers also expressed that the lack of work was affecting their mental health and sense of self. Kayla, 20, an unemployed salesperson turned gig-based food delivery worker, explained, “Today I woke up extra early because my anxiety and depression are kicking in, because I don't have a job to go to. I don't feel important.” As Boland (2015, p. 15) noted, unemployment often creates a sense of “empty time,” or an “absence of doing” that lengthens the day and causes intense feelings of boredom and meaninglessness.

For workers who work by the gig, the sense of unknown was especially troubling, leading to what Abigail, 36, unemployed actor, described as a “psychological” dread. “People who have regular jobs, like a corporate job where they’re working from home, they know that they’re going to be working again soon at their regular job. Or even if they’re furloughed they know most likely, there's a 90% chance that they’re going to be hired back,” she said. “But to be stuck at home and feel powerless with that and have no idea when your entire business is going to start up again is really, really terrifying.”

For workers who often must “hustle” for work, especially those who had worked for enough time to get some level of stability and success, the feeling of suspended animation was also accompanied by a fear of moving backwards. For instance, Lakesha, 46, an unemployed music teacher, actor, and singer lamented the impact on her journey as a performer. “I felt like I was on a really good upward trajectory,” she said. “Right when the pandemic hit. I’m like, ‘Damn it.’ Pushes back all the momentum that I’ve been gathering these two years. So hopefully we will get out of this pause and be in the same place where it will be a real ‘pause’ and not something that we have to restart.”

For workers who had previously been impacted by the Great Recession, the fear of long-term consequences was considerable. As Aanya, 35, an unemployed photographer explained, “I am a go-getter, but to be stamped down again, I don't know that I would come back from that and I don't know what my future would be like, if that happened … I can accept that because I’ve taken on this adventure, but to ask me to sacrifice again, might break me …. I am extremely concerned about my future.”

In addition to struggling with their sense of time, respondents also reported that the pandemic meant that their careers and futures had also entered a period of suspended animation. For instance, Rose, 27, a hotel worker, planned to become an assistant teacher in order to decide if she wanted to return to school to get her teaching certification. The high unemployment rate early in the pandemic, partnered with the fears of a long-term recession, contributed to her sense that it would be impossible to get a job in a new field. As she put it:

For me it feels like everything is on hold, there's no point in making any kind of move that is, I don't know, I just kind of can't even think about it … making any kind of move towards something feels just totally unproductive. … I don't know what is going to happen, nobody knows what's going to happen, what's the point of trying to do anything? That's how I feel.

Work by Barnatt et al. (2017) previously found that career trajectories are substantially influenced by feelings of self-efficacy in relation to perceived labor market conditions. Particularly for young people from low-income backgrounds, a lack of resources constrains individuals’ ability to navigate uncertainty about career direction (Arnett, 2000), potentially contributing to the experience of “aimlessness” that undermines future career advancement (Staff et al., 2010).

### Preparation: “Making my Future”

Workers in the “preparation” category often viewed the pandemic as a time to prepare for the return to work, often focusing explicitly on improving their employability. For these workers, the early lock-down days of the pandemic were used to “prepare” for the end of the pandemic and ensure that they could jump back into what they were doing before, albeit with a running start. Many of these respondents were individuals who worked in live performance, such as acting or dance; or were writers, photographers, and others in the creative class (Florida, 2002).

For these workers, many of whom have high education levels and increased workplace autonomy, the days of staying home were devoted to taking classes or working out, activities that they believed would prepare them for the future and potentially increase their employability. Unlike their white collar office worker peers, these face-to-face creative workers were generally unable to work from home, but could fill their days with free online workshops and tutorials. “We’re able to take classes that we weren't able to take part in before because we had to go work a job and had to go to the studio. I’m taking advantage as much as I can,” said Amy, 45, an unemployed dancer, singer, and dance teacher. Josh, 36, an unemployed restaurant worker, echoed the idea that the shut-down was providing him, “an opportunity to build my skills back up without any capital output.” An unemployed artist who had worked in food service for several years until the pandemic shut-down, he was taking a number of coding boot-camp classes that were being offered for free to unemployed workers, in an effort to change careers. “My biggest fear is that—but I also hope at the same time—that the city will be closed down for the next couple of months so that I can really take advantage of this, which is very selfish,” he said. “I’m just trying to make the best of it for myself while I can.”

Workers often spoke about preparing themselves physically to return to work in better condition—literally—than they had been in before the pandemic. “Changing my diet, and build a repertoire, and just being disciplined … Getting up and just getting back into a disciplined routine to be ready,” said Bernice, 64, an unemployed creative director. “Staying on who's doing what, keeping in contact with people so I know. And making my future, what I want to happen in the next six months and year. So I’m doing all that.”

Part of the focus on preparing for the future was the sense that if workers didn't take advantage of the so-called break, it might have negative repercussions. As Michelle, 36, unemployed lighting designer, put it, “I don't know if people are going to be like, ‘What did you do on your coronavirus vacation?’ Whether or not that has an impact on any future hirings.” Calling the pandemic shutdown a “vacation” is similar to how family leave is often dismissed as an unearned break, ignoring the medical concerns, responsibilities and major life changes often associated with the leave (Cech & Blair-Loy, 2014).

Likewise, for Tyler, 33, an unemployed personal trainer and real-estate worker, there was a sense that one needed to remain “at the ready” for work. “I’m going to make sure that I keep my life afloat because you don't want to be blinded when things come back … I don't feel like I have all the time in the world. … I think when we get that go ahead, it may be even harder if you are not afloat.” This sense that even during a time of major social upheaval—a pandemic, the likes of which hadn't been experienced in a century—was still seen as time for work and career development suggests that the employability doctrine (Vallas & Cummins, 2015) and emphasis on personal branding (Gershon, 2017), already highly prevalent among educated workers, may have taken on increased importance.

This focus on “preparation” for a return to work was particularly evident with Marjorie, 46, a freelance travel writer and fashion designer. Within a week of the lockdown, Marjorie began reaching out to contacts that she had in marketing and public relations. “I would schedule phone meetings with them … picking their brain, ‘Have you spoken to the travel publications? What are they looking for? What are they expecting?’ So I have copious notes and it all went into my database, who's taking queries, who isn't taking queries? What are they looking for? What's going to be a trend?” she said. “I then designed what I call a ‘letter of introduction.’ It's really not—I’ve worked with some of these editors—but it's a way of getting my name back out there, ‘Don't forget me! Here's what I’m available for.’”

Marjorie's use of a “letter of introduction,” sent to individuals she had worked with before, and as a way to “get her name back out there,” further supports the idea that for creative workers there was a sense that they needed to prepare for the future, or risk being left behind. As responsibility for socioeconomic outcomes becomes more individualized (Bauman, 2000; Beck, 1992), young professionals and other workers face increasing pressure to stand out from the competition by proactively “branding” themselves as especially competent or enthusiastic employees (Gershon, 2017; Vallas & Christin, 2018). These attempts to appear more motivated than other job applicants can lead workers to settle for lower pay or less desirable positions (Cech, 2021; DePalma, 2021).

### Rest, Reflect, and Reset: “I Didn't Have the Time to Actually sit Down and Think About my Future”

Finally, on the opposite end of the continuum from those workers who were experiencing a sense of suspended animation, are those workers who identified the early days of the pandemic as a period of rest, reflection, and an opportunity to potentially reset. Intriguingly, while most of the other broad types involved both precarious low-wage employees and gig-based workers, this last category was almost entirely populated by workers engaged in restaurant work or gig work. We theorize that while a sense of speeding up has been found in numerous fields and occupations (Snyder, 2016), gig workers and restaurant workers are service-based, paid by the hour or gig, and regularly expected to be actively moving or “hustling” during their entire work period (Ravenelle, 2019a). For these workers, the CARES Act, and the requirement to literally stay-in-place for weeks at a time—partnered with the financial security offered by enhanced unemployment assistance—was often seen as an opportunity to catch their breath. While many face-to-face creative freelancers often identify a sense of “hurry up and wait” that comes with live performance or content creation, gig workers are usually only paid if they are on the go—and thanks to just-in-time scheduling, restaurant workers that aren't “busy enough” run the risk of being sent home early, with a reduction in their pay (Schneider, 2021).

For Adrienne, 42, a dog walker who regularly exceeded 20,000 steps daily, the pandemic meant a physical break and an opportunity to consider her career. “I love my clients,” she said. “I am so thankful for them, but I don't mind not working those hours right now.” Additionally, Brent, 50, a pastry chef, “went from working 80-h weeks to 30-h weeks, 20-h weeks. Everything slowed down to a halt.” Single and without children, Brent's new-found free time was mostly spent on catching up with leisure activities. “I went out and bought a bunch of stuff for the house, for my apartment and I work out a bunch of hours a day and I run every day,” he said. “I read a few books that I wanted to read for a while … I binge watched a couple of shows.”

For precarious workers who were also parents, the early days of the pandemic—while stressful and scary—sometimes offered a needed respite from the frantic stress of daily hustling. Teresa, 34, a mother and former PhD student turned part-time tutor, who faced drastically reduced hours at her fast food job, was one such worker. As she explained, the pandemic, “has opened doors for me, because before that, I didn't have the time to actually sit down and think about my future.”

Likewise, for Isabel, 33, also an unemployed restaurant worker with three kids, life pre-covid was “hectic.” After her partner would go to work, she would cook, clean and drop the kids off at school. “Before 2:00, everything was ready, and then I have to get ready and go pick up the kids, bring them to the house and then wait until like 3:00, so we can just rest a little, and then I would walk to the train station and then he will be coming out of the train. I give him the kids and I go to work and then he comes back home,” she said. “Then I work and I finish at 10, that was when we finished working; [closing] work and everything, 11, I would get home at 12 midnight, and then yeah, just go to sleep and then do the same next day for five days a week. … It was always in a rush, I was very stressed.” Unlike the unemployed workers who experienced boredom (Boland, 2015), for these low-wage precarious workers who were always on the go, the pandemic provided a needed respite. As a result, these workers who were able to maintain “temporal agency” by structuring their own schedules, perceived the pause as a deeply significant or personally-beneficial experience (Nielsen et al., 2021).

While many of the workers expressed a sense of suspended animation or focus on preparing for the road ahead, workers who reported taking the time to reflect were among the minority in the Phase I interviews conducted early in the Covid-19 pandemic. However, as the pandemic stretched on into the fall of 2020 and winter of 2021, more and more workers began to emphasize taking the time to reflect, resulting in “pandemic epiphanies,” and triggering career changes.

### Phase II: An Increase in “Contemplation” and Resulting Career Changes

The Phase II interviews were conducted between November 2020 and June 2021, coinciding with the second surge of the virus in New York, and with the early days of the vaccine roll-out for adults. Whereas respondents in the preliminary interviews expressed a sense of suspended animation, future-planning, or taking advantage of the shutdown as a time for rest and reflection, in the Phase II interviews there was a strong sense that the pandemic had led to an increased sense of “contemplation,” leading many respondents to report a gradual process of coming to a “pandemic epiphany,” and altering their careers accordingly.

We want to caution here that our use of the concept “epiphany” is not intended to indicate a sudden “eureka” moment of discovery, but can come in different forms, and is the result of significant work. Berkun (2010, pp. 8–9) compares an epiphany to putting the last piece in a puzzle: noting that the last piece of a puzzle has no more magical meaning than the first piece; it's the final result—the finished puzzle—that is the goal. Csikszentmihalyi (2013) describes an epiphany as an extended process including preparation, incubation, and insight. Much like putting together a puzzle can be time-consuming, this reconceptualization of work is a gradual process of valuing one's time in a more personal way, and one that ultimately results in change. The slowing down that occurred during the pandemic enabled the pandemic epiphany: removed from the chaos of pre-pandemic daily life, workers were freed to engage in a new kind of future projection and envision a different trajectory for their lives.

### Contemplation: “Think About Other Alternatives”

As noted previously, restaurant workers and gig-based workers were more likely in the preliminary interviews to describe themselves as using the pandemic shutdown to rest, reflect, and reset. For a number of workers, this time to rest and reset led them to jobs that they saw as offering respite from hustling. For instance, Cameron, 29, transitioned from TaskRabbit, where he specialized in moving gigs, to training to be a yoga instructor, an occupation at the other end of the hustle spectrum. As he explained, “It's definitely made me reconsider and think about other alternatives, and it definitely allowed me to slow down. And so I refocused.” The reference to speed—“to slow down”—further reinforces the experience of time shifting from frivolous expenditure to careful investment. Additionally, taking the time to “reconsider” other alternatives further suggests taking the time to make a larger investment in the self.

Workers also used the pandemic to “invest in the self” and reconsider their current jobs in regards to safety, stability, and security. During the first wave of interviews, 37-year-old Misty was a grocery store cashier, a job that she was understandably nervous about during the pandemic. By the Phase II follow-up interview, she had changed jobs and was working a semi-remote office job for a real estate firm. “I wanted to take my time and choose what I wanted to do next, be more cautious in terms of my next job position,” she said. “I didn't want to be in just any job. I wanted somewhere where I knew I was going to be safe or comfortable, or at least not some dead-end bullshit job.”

She explained that the pandemic had made her “take a step back and reanalyze” and “recreate myself as a person.” Concerned about the job losses that had occurred during the pandemic, she was focused on security and wanted a stable job with benefits and retirement, “where I won't be let go any day at the drop of a dime.” As part of this reanalysis of herself and her career prospects, she was thinking about returning to school, or learning a new language in order to “better market” herself. As she explained: “So Covid has definitely kind of forced me to reinvent myself.” Misty's comments vividly demonstrate the intimate conversion of time into a personal resource: by taking “a step back” to contemplate her life, she realized she must now “recreate myself as a person.” Although Misty drew on the discourse of the “belabored self” (McGee, 2005) in describing her job search as a process of self-invention, she did so to express a newfound unwillingness to work a “dead-end bullshit job” in dangerous conditions. Far from understanding self-improvement solely as a means to increase her employability, Misty experienced the call to “recreate” herself as a personal injunction to hold employers to a higher standard and become more selective about the types of jobs she would accept.

Misty's experience of reflection leading to a sense of work selectivity was not unique. The experience of investing time into personal growth or self-understanding also led Curtis, 32, an unemployed teacher and former marijuana delivery worker, to invest his time in other pursuits more deliberately. “I’m a big believer in contemplation, observation. […] And I don't really think we ever will totally go back to normal, as I said, but I think it will affect everything I do,” he said. “It’ll put much more intent into every stroke of the pen, so to speak, going forward. It already has. So for that, I’m grateful.” Cameron, who left gig work for yoga training, also explained the change as part of a new motivation to follow his passions: “I just don't want to do something that I don't want to do anymore. That I’m not a hundred percent passionate about, and I don't want to just get stuck,” he explained, noting that he now had time “to work on my other passion projects,” including making sugar-free ice cream. Rylee, 33, unemployed voice-over actor, theater worker, and tutor, explained, “You have to look out for your best interest, and I think because we’ve had to do that all year … I’m not going to waste my time on something that I don't really want to do.” It's important to note that Rylee explicitly draws a connection between the experience of attending more to the self during the pandemic (“you have to look out for your best interest …”) and investing, or not wasting, time.

For some workers, this self-reflection and growing sense of selectivity led to a wholesale career change, including new entrepreneurial ventures, as the next section will highlight.

### Pandemic Epiphany: “Is this Something that I Really Want to Do?”

While the pandemic continued during the second wave of interviews, the period of contemplation had led workers to reconsider careers and plot new directions for themselves based on those realizations, a gradual awareness and resulting change that respondents experienced as a biographical turning point and which we describe as a “pandemic epiphany.” Workers were explicit in linking their career changes to the pandemic. For instance, Marissa, 25, an unemployed server and performer discovered that it was important to feel like she was “making a difference” and to know that the work she was doing was fulfilling for herself and others. “I don't think any of this would have even mattered if it weren't for the pandemic ‘cause I think I would have kept doing the same thing I was doing,” she said. “It's a better place for me to be as a worker and I’m happy for it but it's definitely 100% because of Covid.”

The self-reflection opportunity that arose from the pandemic was largely seen as positive. “The pause in our lives has been really great for realizing that that's important and that will make my overall life better,” said Kelly, 29, an unemployed stage manager and lighting designer. For Kelly, realizations about what was important involved creating a bullet journal list of things she wanted to change or work on when the pandemic ended. As part of her epiphany, Kelly also began prioritizing spending time with family, taking “pauses” and “saying no to projects that sound sketchy or I’m not passionate about.”

For many workers, the reflection and resulting decisions to be more selective about work was a novel experience. As Nicole, 52, restaurant worker, explained, “I think for the very first time in my life, I asked myself ‘is this something that I really want to do? Is this something I really like?’” Likewise, for Rylee, the pandemic led to the decision that she will no longer take “anything that isn't paying a fair rate” or “something that isn't … something I’m proud of.”

As part of deciding what they “really like,” respondents also focused on the pursuit of their passions or larger purpose in life. Beverly, 59, was one such worker. Previously a fashion stylist at a local boutique, the pandemic led to her pursuing a career as a substance abuse recovery coach.

It feels wonderful. It feels purposeful … It's not like chasing Chanel or chasing Louis Vuitton. And my new mantra is kindness is the new Chanel. So I’d rather replace my luxury way of thinking for internal beauty and helping people. […] My perspective changed. And I had to really get purposeful.

Beverly's new mantra—“kindness is the new Chanel”—illustrates the change that her time is no longer purely about conversion into income, but reflects a substitution with personal values. Additionally, the need to “get purposeful” with her time further demonstrates a link between investment (purposeful as opposed to frivolous) and personal growth (purposeful as opposed to empty), further highlighted by the fact that she had “outgrown” her previous occupation. Likewise, Spencer, 29, a graduate student, adjunct college instructor and Instacart/Shipt worker who uses they/them pronouns, noted that the pandemic had “clarified” things, describing it as a “catalyst motivation.” Spencer experienced the investment of time into the self as a “catalyst” that set into motion a plan to leave academia. “It's emancipatory to me that I’m doing this career change,” they said, suggesting a level of bondage involved in their previous work. Although workers like Beverly, Spencer, and Cameron borrow heavily from the discourse of work passion to describe their experiences, their appropriation of the passion principle is novel: Rather than reproducing the instrumentalization of passion as a route to (precarious) employment, their pursuit of passion carries aspirations for better, more meaningful, and more dignified work than they had previously endured.

In the most extreme cases, respondents didn't just identify a change in jobs, but also decided to pursue entrepreneurship or self-employment to better align their values and work. Colton, 21, a health spa manager found himself wondering “what value there is to my life” if he spent more time writing emails than enjoying his craft and writing. Colton's contemplation led him to purchase a programmable sewing machine that he used to start a business selling embroidered clothes on Etsy, a change that he directly linked to the pandemic. “I think pre-pandemic, I would have been fine, not necessarily fulfilled, but not unhappy or uncomfortable with the idea of sitting behind a desk in a tie for eight hours, sending emails and stuff like that. Whereas now I feel this pressure to really actually fulfill myself,” he said. “The pandemic has put me in a position where I really want that entrepreneurial mindset. And I want to cultivate something that serves as both a career and just a fulfilling entity for me. I don't want to settle.” For Colton, this “entrepreneurial mindset” applies both to money, through investing money into a long-term enterprise, and to his time, by investing his time in personal fulfillment.

## Conclusion

A historic number of workers quit jobs in 2021, and the US Labor Department reported that a record-breaking 4.3 million workers left their jobs in the month of August alone (Tharoor, 2021). Dubbed the “Great Resignation” by news media and economists, this phenomenon has attracted significant attention from scholars, policymakers, and the popular press and has been linked to ongoing challenges in economic recovery (Cook, 2021; Tharoor, 2021). In particular, restaurants, bars and hotels (6.8%) and retail (4.7%) had some of the highest quit rates as of August 2021; 38 percent of the workers who quit in August 2021 worked in retail or in hotels and restaurants (Rosenberg et al., 2021). The unprecedented pace of resignations has been attributed in large part to self-reflection and the desire for better or more fulfilling employment opportunities (Tharoor, 2021).

In this paper, we ask, how has a changing conception of free time during the Covid-19 pandemic affected precarious and gig-based workers? In answering this question, we draw on a mixed methods panel study of 199 precarious or gig-based workers in the New York metropolitan area, conducted early in the pandemic and during the second surge.

We find that during the initial outbreak of Covid-19, respondents reported that their overabundance of free time was experienced as a disruption to the conversion of time into money, and the main problem facing most respondents was how to expend time in a way that continued to generate income. Workers often broadly fell into one of three broad categories: suspended animation; preparation; or rest, reflect, and reset. While worker classification did not appear to impact the number of workers who identified as experiencing “suspended animation” or “preparation,” those in the rest and reset category were more likely to be restaurant workers and gig-based workers.

During the second surge of the pandemic, respondents reported using surplus time—the time they previously converted into income prior to the pandemic—on various kinds of contemplation and self-improvement. As respondents’ experiences of time changed from “spending time” making money to “investing time” in themselves, so did their perceptions of time: a use-mindset gave way to an investment-mindset as respondents came to value time as a resource that initiates various kinds of personal growth. In the Phase II interviews, there was a strong sense that the pandemic had led to an increased experience of “contemplation,” leading many respondents to a “pandemic epiphany,” and altering their careers accordingly, with considerable career changes, including entrepreneurship.

As a result, following an initial period of hysteresis when instrumental understandings of time were disrupted by lockdowns that prevented the normal conversion of labor time into wages, financial security from unemployment benefits and stimulus checks may have enabled workers to gradually adopt a new conception of time as a resource for personal growth. Later, greater stability and acclimation to pandemic conditions enabled respondents to increasingly perceive time as an investment in personal growth, yielding commitments to seek better and more fulfilling work. This shift from instrumental rationality to contemplative self-improvement in participants’ understandings of time is primarily expressed through the adoption of a prevailing “passion principle” (Cech, 2021) or “work passion” ideology (DePalma, 2021) that inspired major career changes in pursuit of self-affirming work.

Although our respondents often expressed interest in work that remains precarious, like yoga instruction, these jobs might nonetheless provide higher wages and more stability than gig work, in addition to greater personal fulfillment. In addition, many respondents aspired to find more secure and better-paying jobs, often affirming commitments to become more selective in the types of work they would accept. These workers did not understand the pursuit of passion primarily in terms of increasing their employability; instead, the desire to find meaningful work was expressed as an outcome of their increased self-worth. Our findings extend previous research that documents adherence to the passion principle among educated professionals, including during the pandemic (Cech & Hiltner, 2022), by demonstrating that many low-wage and gig workers have also adopted it as an interpretive frame for their recent experiences.

More work needs to be done to examine how and why these workers reinterpreted the passion principle to define turning points in opposition to employability, but two major factors distinguish our sample from workers in previous studies. First, our participants benefited from an exceptional degree of financial security through unprecedented financial assistance from the U.S. government. Second, as low-wage precarious workers, our participants typically lacked prior expectations of secure or high-paying employment that are exhibited, at least initially, by white-collar professionals in previous studies (Lane, 2011; McGee, 2005; Snyder, 2016). The financial assistance provided through the CARES Act and multiple stimulus checks may have enabled these highly precarious workers to benefit from personal sites of hyperprojectivity that supported a transformative reorientation toward the future as their schedules, concerns, and responsibilities were suspended or mitigated.

That said, as the historic degree of financial support has ended, commitments to seek personal fulfillment may have already been discarded or realigned with the goal of employability. It is still unclear how many participants who expressed intentions to seek better jobs will ultimately follow through, and career changes might fail or land workers in even more precarious jobs. These experiences of unemployment could cease to be defined as turning points in retrospect, becoming instead a temporary lull from the difficult reality of precarious low-wage work. On the other hand, our study documents a novel reconstruction of work passion into a biographical turning point and rejection of employability among highly vulnerable workers, suggesting that some aspects of this discourse—like its emphasis on self-determination—may hold greater progressive potential than is typically acknowledged (see McGee, 2005, p. 162).

Additional work is needed to examine the long-term impacts of the pandemic on the career paths of precarious and gig-based workers, and to study the longevity of pandemic-inspired entrepreneurial efforts. In our ongoing Phase III interviews, we are examining the extent to which participants continue to express their plans in terms of work passion ideology, and the prevalence of career changes made in pursuit of more personally fulfilling work.

Our findings demonstrate that work passion ideology has been adopted by workers outside the creative industries, and that the pursuit of passion among low-wage precarious workers can inspire these workers to actively search for more fulfilling and potentially lucrative jobs. Previous research has found that work passion ideology acts as an individualizing cultural frame that encourages professionals to tolerate unstable and low-wage work in artistic or media-related fields (Adler, 2021; Cech, 2021; DePalma, 2021). Among precarious service and gig workers, however, the embrace of the passion principle may be contributing to a rejection of stressful and poorly compensated service jobs. This study shows that in addition to promoting acceptance of unstable employment, work passion ideology can motivate resistance, suggesting the need to further examine its role in the reproduction of economic precarity.

## Notes

1. The creative freelancers that we include in this sample worked in person as actors/extras, dancers, models, photographers, and film crew staff—jobs that had limited potential/demand for online services. For the workers we interviewed, these jobs did not offer a consistent income, generally did not offer benefits, were short-term (a modeling gig or photo shoot would generally last a few hours, an actor might only have work for a day or week), and rarely offered remote options.

2. While this range of $40,000 to $70,000 may seem high, according to U.S. Census Bureau’s 2013–2017 American Community Survey 5-Year Estimates, the median individual income in New York City is $50,825. The New York City Housing Authority (NYCHA), which provides “public housing” for low and moderate income residents considers single residents eligible for subsidized housing if they made $74,800 or less annually in 2022 (https://www1.nyc.gov/site/nycha/eligibility/eligibility.page).
